# The First Case of Infectious Spondylitis Caused by *Gemella bergeri*

**DOI:** 10.3390/medicina59010145

**Published:** 2023-01-11

**Authors:** Kyoung Ree Lim, Jun Seong Son, Soo-youn Moon

**Affiliations:** Division of Infectious Diseases, Department of Internal Medicine, Kyung Hee University Hospital at Gangdong, Seoul 05278, Republic of Korea

**Keywords:** *Gemella bergeri*, bacteremia, infectious spondylitis

## Abstract

*Gemella bergeri*, a member of the genus *Gemella*, is a facultatively anaerobic, Gram-positive cocci. *G. bergeri* is a component of normal oral flora; however, it can become pathogenic and cause infections in patients with poor oral hygiene. A 78-year-old man was admitted to a hospital with a complaint of increasing posterior neck pain and lower back pain for 2 weeks. MRI was suggestive of infectious spondylitis at the C3-C4 level with prevertebral abscess formation, anterior epidural abscess formation. We identified *Gemella bergeri* in closed pus obtained during the surgery. Herein, we describe the first case of infective spondylitis caused by *G. bergeri*.

## 1. Introduction

*Gemella bergeri* is one of nine species (the others include *G. haemolysans*, *G. morbillorum*, *G. sanguinis*, *G. asaccharolytica*, *G. taiwanensis*, *G. parahaemolysans*, *G. cuniculi*, and *G. palanticanis*) belonging to the genus Gemella. It is a component of the normal flora of the oral cavity, digestive tract, and urinary tract [1,2]. It was first isolated by Collins et al. in 1998 [3]. The organisms belonging to the genus Gemella rarely cause systemic diseases; however, like other commensal bacteria, they can cause opportunistic infections, especially in immunocompromised patients. To our knowledge, cases of infectious spondylitis caused by *Gemella bergeri* have not been reported so far. Herein we describe the first case of infectious spondylitis caused by *G. bergeri* in Korea.

## 2. Case Presentation

A 78-year-old man was admitted to hospital with a complaint of increasing posterior neck pain, lower back pain, and both shoulder pain for 2 weeks. His past medical history included type 2 diabetes mellitus, hypertension, and old myocardial infarction, for which he was taking medications. The patient was treated with physiotherapy and pain control medications for posterior neck pain.

**Figure 1:**
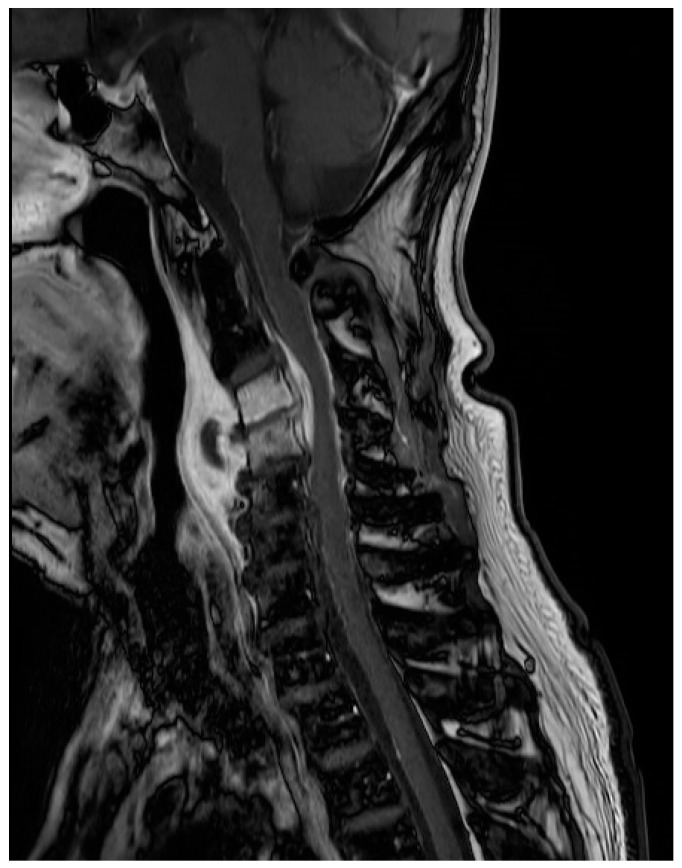
Magnetic resonance images showing infectious spondylitis with prevertebral abscess formation and anterior epidural abscess formation at the C3-C4 level, and a diffuse prevertebral soft tissue swelling/inflammation.

On admission, his height and weight were 164.3 cm and 52.7 kg (body mass index (BMI): 19.52). He was alert, and his vital signs were stable, with a blood pressure of 132/81 mmHg, heart rate of 80 beats/min with a regular rhythm, and body temperature of 36.9 °C. Laboratory data at the time of admission showed an erythrocyte sedimentation rate (ESR) of 102 mm/h (normal range: ~15 mg/dL) and C-reactive protein (CRP) level of 5.9 mg/dL (normal range < 0.5 mg/dL). The white blood cell count was 12.9 × 10^3^/μL (normal range: 4 to 10 × 10^3^/μL). MRI was suggestive of infectious spondylitis at the C3-C4 level with prevertebral abscess formation (about 2 × 0.9 × 1.4 cm in size), anterior epidural abscess formation (about 1.6 × 0.2 × 0.7 cm in size), and a diffuse prevertebral soft tissue swelling (Figure 1).

A two-stage operation was performed. The discectomy was performed at C3-C4 level of the spine with irrigation on the day after admission. The culture specimens were obtained from soft tissue at C3-C4 and epidural abscess in operating room during the surgery. The antimicrobial therapy was initiated with vancomycin and ceftriaxone before the culture results became available.

On the fifth day of hospitalization, a second operation was performed to insert the fusion device.

**Figure 2:**
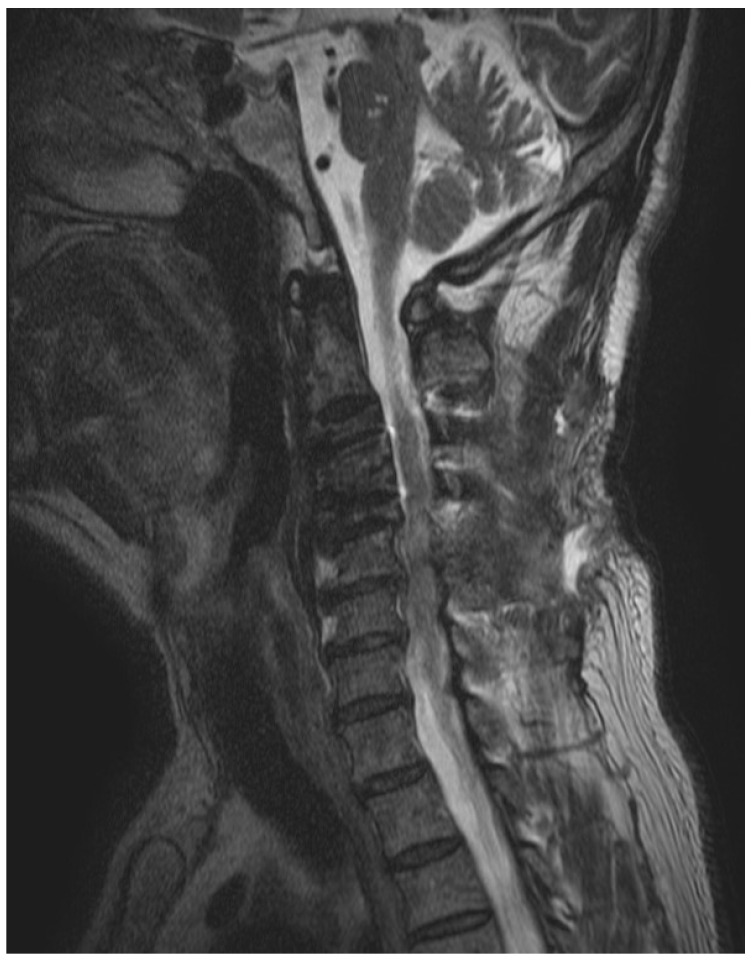
On follow-up magnetic resonance images, there were no previously observed prevertebral abscesses.

A blood culture was performed before initiating the empirical antimicrobial treatment. However, no pathogens were identified in blood cultures. *Gemella bergeri* was identified from a specimen obtained during the first surgery. Matrix-assisted laser desorption ionization–time-of-flight mass spectrometry (MALDI-TOF) (bioMerieux, Marcy-l’Etoile, France) was used to identify causative pathogens. Additionally, the antibiotic susceptibility test was performed with the VITEK-2 system. An antibiotic susceptibility test reported susceptibility to penicillin, clindamycin, and vancomycin. After the pathogen was identified, the empirical treatment was changed to ampicillin-sulbactam on the ninth day of hospitalization. Intravenous ampicillin-sulbactam was continued for 9 days before the patient was discharged from the hospital. The antibiotic regiment was then switched to oral amoxicillin/clavulanate. After 2 months of treatment, a follow-up MRI was performed, and no previously observed prevertebral abscesses were noted (Figure 2). Laboratory tests showed an ESR of 17 mm/h and a CRP concentration of 0.1 mg/dL. The antibiotic treatment was discontinued. At a follow up visit performed 2 months after discontinuation of treatment, the patient’s symptoms had improved.

## 3. Discussion

Gemella species are believed to be harmless microorganisms in the upper respiratory, gastrointestinal, and genitourinary tracts. They are a part of the normal oral flora and frequently found in patients with white spot lesions and gingivitis [4]. *G. bergeri* is one of the nine species of the genus Gemella and comprises catalase-negative, facultatively anaerobic, Gram-positive cocci occurring in pairs, tetrads, and/or short chains [5].

When using conventional biochemical methods, *G. bergeri* can be misidentified as other Gemella species or viridans streptococci. Thus, identification of *G. bergeri* is relatively difficult [6]. In 1998, it was first isolated from a blood cultures by Collins et al. Six patients were reported, three of whom had bacterial endocarditis [3]. The number of reported cases has gradually increased since then. According to a case reported by Toyoshima et al. in 2021 [7], 15 cases had been reported by the time their case was reported. The previously reported cases included cutaneous orbital abscess, Lemierre’s syndrome, and meningitis; however, the majority were infective endocarditis (IE) [1,2,3,6,8,9,10,11,12].

IE is often caused by bacteria that colonize teeth. It is known that bacteria may enter the bloodstream when the patient has poor oral hygiene or gingival bleeding. Therefore, bacteremia may occur after toothbrushing or dental procedures. A previous study showed the relationship between oral hygiene and gum disease and the risk of developing IE-related bacteremia after daily activities, such as toothbrushing [13].

Infectious spondylitis accounts for approximately 3–5% of all osteomyelitis cases yearly, and its incidence has increased in recent years. Infectious spondylitis often presents as a non-specific clinical condition; therefore, its diagnosis is often delayed [14,15]. Infectious spondylitis can usually occur through the following routes: hematogenous spread from a distant site or focus of infection, direct inoculation, or contiguous spread from adjacent soft tissue infection [15].

Our patient had a history of recurrent periodontitis, and the patient received regular check-ups every 3 months at a dental clinic. The patient did not have bacteremia at the time of diagnosis. However, the route of infection can be estimated by considering a history of recurrent periodontitis. It can be assumed that transient bacteremia occurs first, followed by infectious spondylitis, through the hematogenous spread of infection.

To our knowledge, infectious spondylitis caused by other Gemella species has been reported [16,17]; however, infectious spondylitis caused by *Gemella bergeri* has not been reported so far.

As described above, *G. bergeri* is a normal oral flora, so careful interpretation is required when this organism is identified in culture studies. When evaluating an identified organism as pathogen, the collecting method of the specimen and the clinical information of the patient should be considered. It can be a pathogen that can cause infections in patients with dental problems and poor oral hygiene. Herein, we report a case of infectious spondylitis caused by *G. bergeri*.

## Data Availability

Data is contained within the article.
